# Utilising random forests in the modelling of *Eragrostis curvula* presence and absence in an Australian grassland system

**DOI:** 10.1038/s41598-023-43667-w

**Published:** 2023-10-03

**Authors:** J. Brown, A. Merchant, L. Ingram

**Affiliations:** 1 The University of Sydney, Sydney, Australia; 2 NSW Department of Primary Industries, Queanbeyan, Australia

**Keywords:** Ecology, Ecosystem ecology, Grassland ecology, Invasive species

## Abstract

*Eragrostis curvula* is an agronomically and ecologically undesirable perennial tussock grass dispersed across Australia. The objective of this study is to investigate relationships of ecologically relevant abiotic variables with the presence of *E. curvula* at a landscape scale in the Snowy Monaro region, Australia. Through vegetation surveys across 21 privately owned properties and freely available ancillary data on *E. curvula* presence, we used seven predictor variables, including Sentinel 2 NDVI reflectance, topography, distance from roads and watercourses and climate, to predict the presence or absence of *E. curvula* across its invaded range using a random forest (RF) algorithm. Assessment of performance metrics resulted in a pseudo-R squared of 0.96, a kappa of 0.97 and an R squared for out-of-bag samples of 0.67. Temperature had the largest influence on the model’s performance, followed by linear features such as highways and rivers. Highways’ high importance in the model may indicate that the presence or absence of *E. curvula* is related to the density of human transit, thus as a vector of *E. curvula* propagule dispersal. Further, humans’ tendency to reside adjacent to rivers may indicate that *E. curvula*’s presence or absence is related to human density and *E. curvula*’s potential to spread via water courses.

## Introduction

*Eragrostis curvula* is a species that has attracted government, public and scientific interest due to its impacts on ecological integrity and agricultural productivity^1–3^. *E. curvula* is a C4 perennial tussock grass in the family *Poaceae*. The *Eragrostis* genus has 300 species worldwide, with 66 present in Australia, 52 native, and 14 considered non-native species^4^. *E. curvula* was intentionally introduced into Australia as early as 1910 by the Australian Government as part of a larger scheme to introduce soil-stabilising plant species that may also offer a source of feed to grazing livestock^5,6^. In Australia, *E. curvula* has become an undesirable plant species in agricultural and ecological contexts, impacting species diversity, richness, and agricultural productivity^7,8^. However, evidence suggests that *E. curvula* can sustain agricultural enterprises but requires intensive management to be a moderate pasture grass, regarding sustaining livestock health and weight for productive agricultural output while still impacting native species diversity^5,6,9,10^. Since its introduction, *E. curvula* has spread and is now present in every state in Australia, including high densities in agricultural regions^7,8,11^.

Factors driving *E. curvula’*s ability to distribute so widely in Australia and across the world include its high propagule pressure and phenotypic plasticity, allowing it to establish itself in a wide range of climatic conditions^6,10,12–14^. The extent to which a species can alter its morphological or physiological traits to suit various landscape conditions defines the amount of phenotypic plasticity a species possesses^14–16^. Firn et al.^14^ found *E. curvula* to have greater variability in six measured plant traits when growing conditions were altered compared to two other co-occurring native species (*Eragrostis sororia* and *Aristida personata*), indicating that *E. curvula* has greater phenotypic plasticity. However, this phenotypic plasticity has been shown to act as a double-edged sword; where when nutrients are applied to *E. curvula*, it will increase its resource use efficiency, thus absorbing nutrients desirable to foraging organisms and becoming a more desirable forage for herbivores and subsequent decrease in *E. curvula* abundance^14^. The findings by Firn et al.^14^ support the ability of *E. curvula* to adapt its phenological development to a wide range of conditions but also offer unique insight into potential management strategies. More broadly, *E. curvula*’s continent-wide distribution may result from its ability to adapt to various stressful and varied environmental conditions^16^. This phenotypic plasticity appears common across cultivars of *E. curvula* and is reflected by relatively distinct yet variable properties such as appearance palatability and phenology within the species^17,18^.

Holmes^19^ reported on the impact of both the perennial weeds *E. curvula* and serrated tussock on the Monaro grazing industries suggesting that the difference between the cumulative cash surplus of an uninvaded farm and an invaded farm can be up to $300 000, primarily due to the difference in return from livestock. In an invaded farm situation, the land’s carrying capacity can be as low as two dry sheep equivalent (dse) per hectare, which according to Holmes^19^, is insufficient to “break-even” and results in an income deficiency. Holmes^19^ categorises uninvaded native pastures as being able to sustain four des per hectare and eight des per hectare in modified pastures. Holmes^19^ argues that the benefits of managing invasive plants, despite the monetary cost of labour and resources required, far outweigh the financial cost impacts of no management due to invasive species’ impact on an area’s carrying capacity decreasing a farm’s gross income.

Due to its ability to impact both native ecosystems as well as economically important grazed grasslands, research has been conducted that relates the ecological factors that influence the distribution of *E. curvula* and which may be integrated into a single model used for the prediction of distribution across larger spatial areas^6,10,20^. Predictive models are dependent on relevant predictor variables, and in the case of species distribution models, understanding what predictor variables are most likely to influence a species distribution is vital in the development of accurate models^21–23^. Thanks to developments in the accessibility of open-source data through data collectors such as Copernicus, Earth Explorer, and the Bureau of Meteorology, predictive models have become more accurate and widespread^24,25^. *E. curvula* is considered an invasive species in Australia, among other countries, and predictor variables influencing its distribution relate to its invasive traits, such as the production of many small seeds and the ability to grow under a wide range of climatic conditions^3,6,10^. *E. curvula* is primarily dispersed by hydrochory (water dispersal), anemochory (wind dispersal) and zoochory (animal dispersal), and as such, predictor variables relating to climate, distance to waterways and distance to human and animal dispersal pathways (such as roads) are likely to be the strongest predictors of *E. curvula* distribution^6,10^.

Random Forest models (RF) are a highly flexible and robust machine learning technique that has gained prominence in various fields of study, including research on drought, land-use change and predictive species distribution modelling^26–31^. Random forests are an ensemble classifier formed from many classification and regression trees^26^. Roozbeh Valavi, Elith, Lahoz‐Monfort, and Guillera‐Arroita (2023) highlight the advantage of flexible models, such as RF, in their ability to generalise data and model complex, non-linear relationships. An issue often present in machine learning techniques is overfitting^26^. The inherent model architecture of RF, such as the incorporation of bootstrapping, random feature selection and the model’s ensemble mechanisms, work to overcome overfitting issues^26^. However, RF are not immune to overfitting data issues, and appropriate performance metrics analysis is required to evaluate RFs^26^. Jalayer et al.^27^ use RF and a neural network architecture for supervised learning, fussy ARTMAP, to model land cover change in the Chalus Watershed, Iran. Using these models, Jalayer et al.^27^ provide evidence of increases in agricultural land and barren areas and declines in grasslands and forested areas where the major land use changes between 2001 and 2021. Further, the model’s prediction was validated on the known land use change and found to be an accurate simulation, with K-index values ranging from 0.92 to 0.94. Here, the K-index values approaching one, reflect a high degree of accuracy between the predicted and actual land use change maps. Jalayer et al.^27^ used these models to forecast land use change into 2040 and predict a further decrease in forest cover and increase in expansion of barren areas, agricultural land and build-up area.

There is a general consensus among invasion ecologists that simply eradicating invasive plants is not feasible in landscape management of social, environmental, and economic values where a species has been established for many years and has developed substantial aboveground biomass and soil seed bank and replaced native species that fulfil a similar role in the ecosystem^32,33^. Rather, mitigation of impacts on the most susceptible areas and preventing the further spread into uninvaded areas is the true goal, especially for species established for decades, such as *E. curvula*^32,34^. With an overarching goal of limiting the spread of invasive species into uninvaded areas, our research objective is to investigate the extent of easily accessible abiotic variables’ ability to predict the presence or absence of *E. curvula* at a landscape scale in the Snowy Monaro region, Australia, using random forest modelling techniques. Random forests (hereafter RFM) are powerful classification and regression methods that function by combining multiple randomised decision trees and aggregates their predictions by averaging^35,36^. We achieve the research objective through observational studies using *E. curvula* in the Snowy Monaro Regional Council area as a case study species and region.

## Methods

### Study area

**Figure 1:**
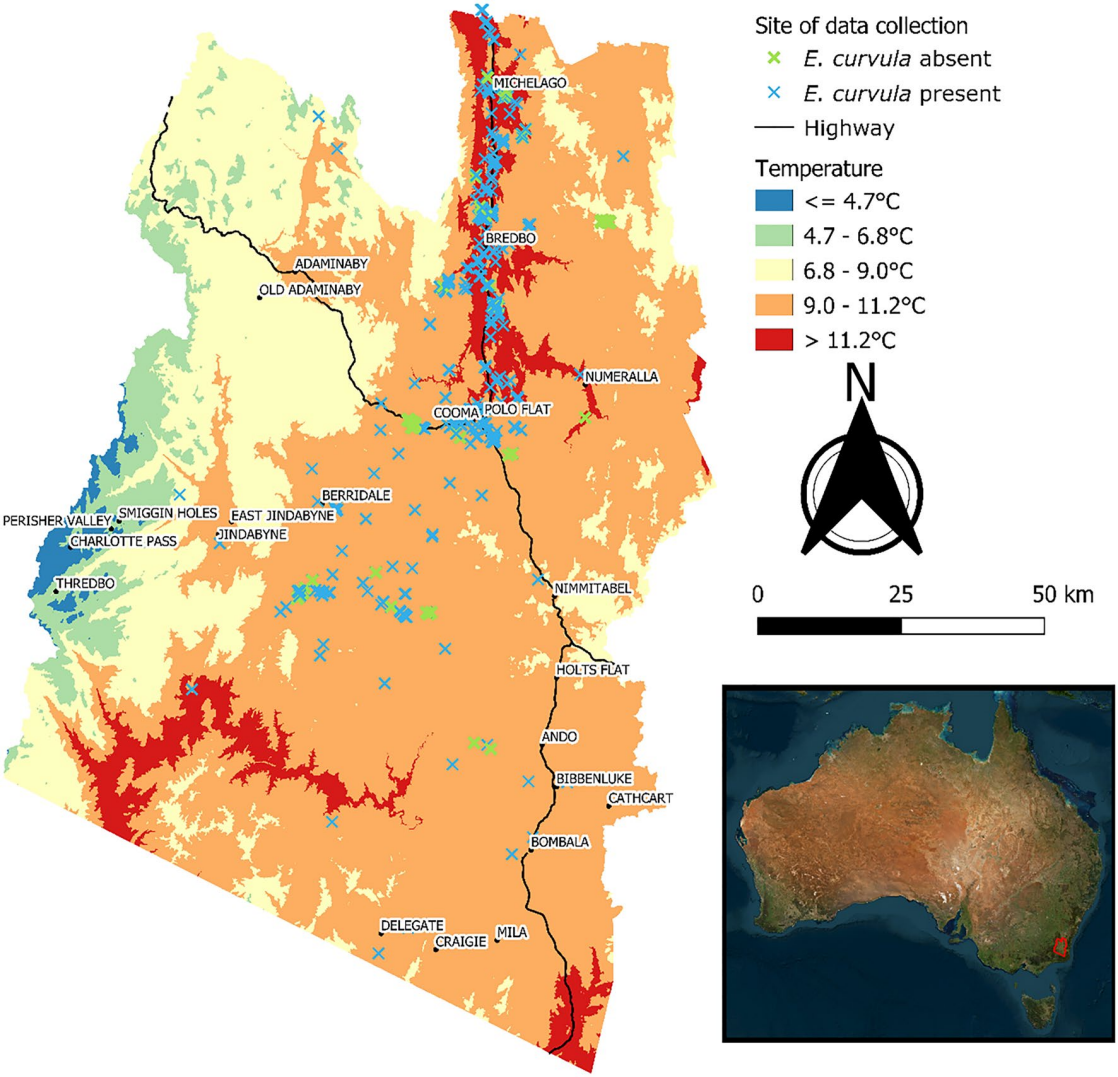
Map of the study locations within the Snowy Monaro Local Government Area. The blue crosses indicate areas surveyed that did not have *Eragrostis curvula* present, while the green crosses indicate areas where *E. curvula* was absent. Temperature (°C) is represented as the annual mean temperature between 1976 and 2005^42^.

The extent of the study area covers the New South Wales Local Government Area of Snowy Monaro Regional Council (hereafter SMRC), which has an approximate area of 1,516,000 hectares (top: −35.579312, right: 149.602741, bottom: −37.262980, left: 148.200678) (Fig. 1). The mean annual temperature within the SMRC between 1976 and 2005, derived from monthly mean temperature data, is 9.4 °C, with a standard deviation of 1.5 °C^37^. The notable rivers of the region are the Murrumbidgee and Snowy, and many more sub-water ways connecting to the river systems. Two major highways run within the SMRC; the Monaro highway runs approximately north–south, and Snowy Mountains highway runs from the northwest, meeting up with the Monaro highway at Cooma, then continues east approximately 9 km south of Nimmitabel, which provides transport of livestock, fodder, residence and tourists. The SMRC ranges in elevation from a minimum of 213 m above sea level to a maximum of 2223 m above sea level^38,39^. The region’s mean elevations are approximately 1000 m above sea level, with a standard deviation of 259.2 m^38,39^. The region’s higher elevations are located towards the east along the portion of the Great Dividing Range commonly known as the Australian Alps and home to Mt Kosciuszko. This study excluded nature conservation areas from potential sites due to the research scope focusing on grazing systems that spatially dominate the SMRC. Excluding nature conservation areas removes large areas of the Australian Alps from the study area and focuses on areas within the Monaro tableland, which is approximately represented by the Interim Biogeographic Regionalisation for Australia^40^, Version 7 sub-region “Monaro”^41^.

### Field method

**Figure 2:**
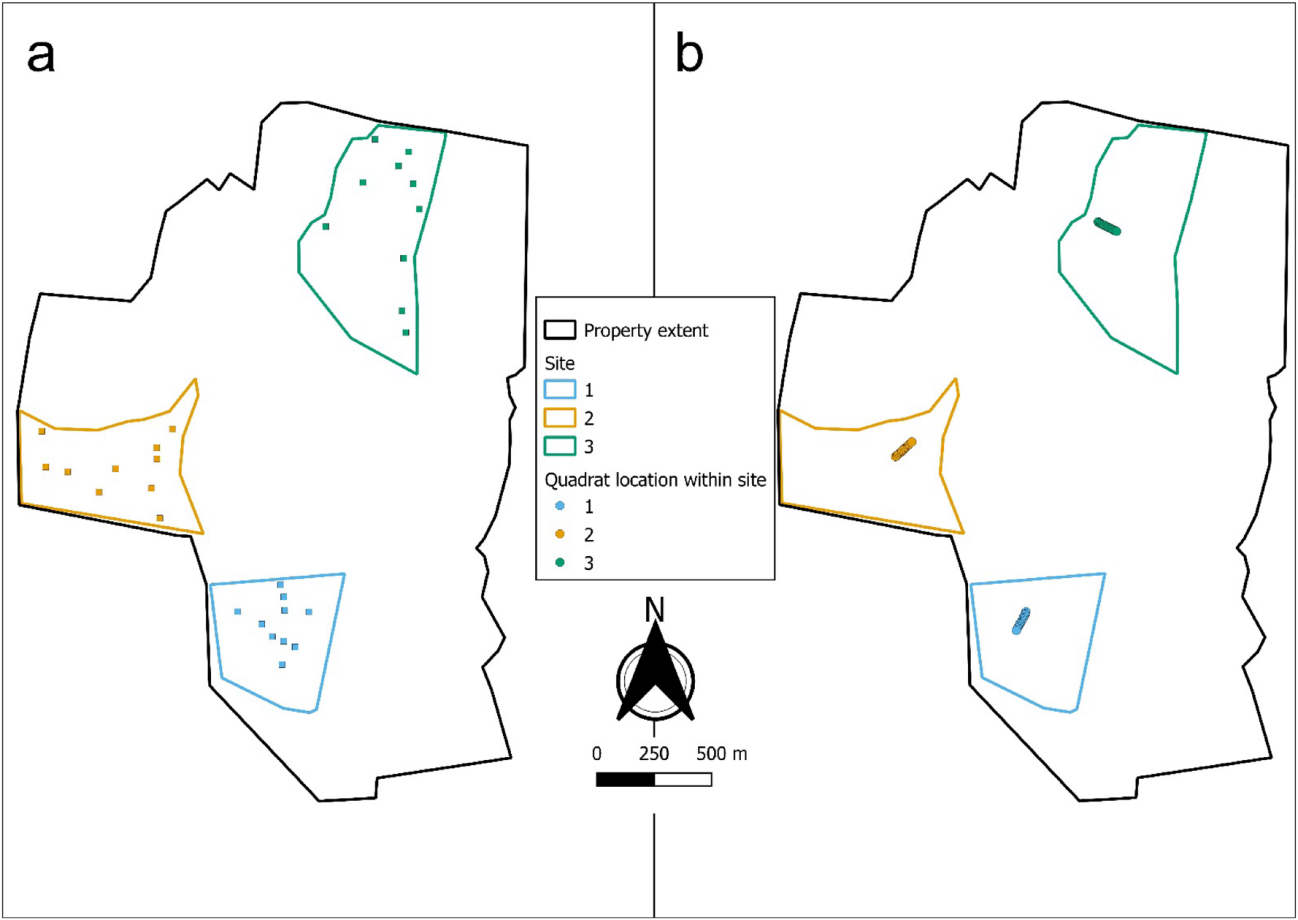
Schematic of the two methods used to collect data for one of the 21 properties used in this study. (**a**) The random points within polygons using the polygon method. Each quadrat in (**a**) is at least 20 m apart from another quadrat. The site polygons are at least 5 hectares in area and were used to bound the random placement of the quadrates within. (**b**) The transect method with quadrate spaced 5 m apart along a 100-m transect. The transects in (**b**) run perpendicular to roads or tracks within the property.

Field data collection occurred from September to December 2019 and again from August to October 2021 (Supplementary Information). The 2019 data collection method involved placing a transect in a location representing the vegetation community of interest and perpendicular to established roads of varying use. Transects were established in areas that contained either high or no/low density of *E. curvula*. Low-density sites are defined as not having any *E. curvula* present, or where *E. curvula* is present; it is not present in swards larger than 10 m^2^. High-density sites are defined as having *E. curvula* in dense swards covering the majority of the sampled site. The transects were 100 m long with 20, 50 × 50 cm quadrats stratified along the length, with GPS points recorded at each quadrat (Fig. 2). The number of replicates at each site depended on the property’s area, with larger properties having more transects, with between 20 and 80 quadrats per property.

The 2021 data collection method differed from that of 2019 in that out of the 21 properties originally sampled, six were selected as sites having a particularly high or low density of *E. curvula* or sites that have areas of high *E. curvula* density in some locations within the property and low density in others. At these sites, three areas of at least five hectares were outlined as a polygon using QGIS software, and ten points were randomly chosen within each polygon, with no points being within 20 m of another (Fig. 2). At the location of each of the points, a 1 × 1-m quadrat was placed, and a vegetation survey was conducted.

### Ancillary data

Due to black summer bushfires across this region during Dec 2019–Mar 2020 and institutional COVID-19 travel restrictions, the data collection on additional properties in 2020 was greatly impeded. Due to these restrictions, a decision was made to use ancillary data in the form of the Atlas of Living Australia^11^ *E. curvula* dataset (Supplementary Information). The Atlas of Living Australia^11^ acts as an *E. curvula* presence dataset with information on location within the Snowy Monaro Local Government Area, bolstering the number of *E. curvula* present data points. The ancillary data was refined to only include records from the year 2000 onwards and had a coordinate uncertainty of less than 100 m.

Copernicus Sentinel data^43^ provide global satellite imagery at spatial resolutions of ten meters for bands 2 (blue), 3 (green), 4 (red) and 8 (near infrared). Bands 4 and 8 can be used for the calculation of NDVI, an index of the greenness of vegetation and a proxy of vegetation density using the formula:

1 $$\frac{(Band\,8-Band\,4)}{(Band\,8+Band\,4)}.$$

The calculation is performed on each pixel within the satellite imagery using the open software provided by QGIS.org^44^. This research utilises Copernicus Sentinel data^43^ collected on 01/11/2019 on sentinel tiles T55HFA, T55HFV, T55HGA and T55HGV. This date was chosen as it falls within the sampling period for this study’s first data collection period. Using the open software QGIS.org^44^, values for NDVI were extracted at each data point using the sample raster values function, resulting in each point used to train the model having a corresponding NDVI value recorded at that location.

Using the open software QGIS (version 3.16.9), the distance (in metres) from each survey location to the nearest road was determined^44^. Roads included all named roads within NSW and farm tracks used to navigate the interior of properties individually added for each property surveyed; in addition, the role that highways may have played as a vector of spread was included in the RF as a separate predictor^45^. Using QGIS (version 3.16.9), the distance from each survey location to the nearest hydroline was obtained using the distance to nearest hub (line to hub) function. The Hydroline data includes surface water features such as lakes, rivers, creeks, canal drains, spillways, races, tunnel-siphons and connectors^46^. The values recorded are to the nearest meter Hydroline was subset only to include rivers, and the same methodology used to calculate the distance to hydroline was used.

Mean annual temperature (°C) and rainfall (mm) data for the period 1976–2005 at the spatial resolution of 1 Arc-second were obtained^37^. One arc second is approximately 30 m but varies due to the earth’s irregular oblate spheroid, or geoid, shape. The mean temperature and rainfall values were extracted at each survey point using the sample raster values function. *Eragrostis curvula* has been suggested to be present in the study area for at least 70 years; as such, it has spread over the area under a changing climate as a result of anthropogenic climate change^47^. As such, this research has chosen to use mean annual temperature data and monthly mean precipitation from 1976 to 2005 as it would best reflect the climatic conditions present in the region during a large time period of its establishment and spread^5,47^. For each site, the elevation (m) above sea level (asl) was determined using a one Arc-second resolution digital elevation model based on data collected in February 2000 Gallant et al.^38^.

### Random forest model

Random Forest creates many (e.g. 500) bootstrap samples, and the data that does not appear in each bootstrap sample is known as the out-of-bag (OOB) sample^35,36^. The bootstrapped samples are passed through a classification tree with randomised binary splits at each node with a random subset of the provided predictor variables^35,36^. The OOB sample from each bootstrap sample is then run through a classification tree, with each OOBs sample classification being recorded, with the final classification of a given sample being the majority vote of all of the classification trees^35,36^. We developed an RFM with binary response variables (hereafter RF P/A) for classifying the presence/absence of *E. curvula* based on seven predictor variables. The predictor variables for the RF P/A are elevation, distance to road, distance to highway, distance to river, distance to any watercourse, Sentinel 2 NDVI, mean temperature over 40 years and mean rainfall over 40 years. All predictor variables are continuous.

The data set for the RF P/A comprises 1292 observations, a combination of in-field measurements using field methods described above, totalling 1096 data points, and *E. curvula* presence data gained from the Atlas of Living Australia^11^, totalling 196 data points. The Atlas of Living Australia^11^ data comprises 196 presence data points. The data set has 612 instances of *E. curvula* absence and 680 instances of *E. curvula* presence.

The RF P/A hyperparameter tuning was conducted using a grid search, where the researcher compared a combination of different hyperparameters chosen to determine the optimal model. As the RF P/A is created for prediction, the metric used to tune the hyperparameters is the error rate or the number of correct predictions the model performs on a data set^48^.

### Statistical analyses

All mapping outputs were created using open-source GIS software version 3.16.9-Hannover produced by QGIS.org^44^. All model calculations were performed in the open-source R software Version 4.2.1 produced by RStudio Team^49^. The model was trained and evaluated using the train function inside the R package Ranger. Metrics to assess model performance are AUC, R squared for the out-of-bag samples (R squared OOB), pseudo R squared, Cohen’s Kappa, sensitivity, specificity and F1 score^48^. Predictor variables of elevation and NDVI were found to be highly correlated (> 0.65) with other predictor variables in the model and thus removed as they had the lowest importance of the correlated variables when included in the RF P/A. Further, the ecological justification of retaining the correlated predictor variables of mean annual rainfall and mean annual temperature relates to the ecology of *E. curvula* as species tolerant of dry conditions but potentially intolerant to certain temperature extremes^6,10,50^. Due to the fluctuating nature of performance metrics when conducting RFM, the optimal RF P/A underwent 30 repetitions, with the performance metric being recorded from each interaction and allowing for a measure of spread using standard deviation. Predictions were generated using the predict function from the inbuilt stats package in R.

## Results

### Presence/absence random forest model performance

The RF P/A for *E. curvula* has an AUC of 0.9995, R squared OOB of 0.67, and a pseudo-R squared of 0.96 (Table 1). The relatively low standard deviation of the model after 30 repetitions indicates the model is performing consistently across the 30 repetitions. The model had a very good agreement regarding Kappa with a value of 0.97, other performance metrics of sensitivity, specificity and F1 scores of 0.98, 0.99 and 0.98, respectively (Table 2). However, as Lantz^48^ notes, the term very good agreement is accompanied by subjectivity. Comparing the pseudo-R squared, AUC and Kappa to the R squared OOB indicate that the model performs with high accuracy when classifying data that it was trained on but lost accuracy when classifying completely new data. Regardless, a random model that arbitrarily assigns points as having *E. curvula* present or absent would be expected to have an AUC of 0.5 and an R-squared OOB of 0, assuming a binary response and equal samples in the presence and absence dataset.

**Table 1:** Performance metrics used to assess random forest performance.

Performance metric	Mean after 30 repetitions	Standard deviation after 30 repetitions
Aera under curve	0.9995	0.00003
R squared (out of bag)	0.67	0.001
Pseudo R squared	0.96	0.0003

**Table 2:** Confusion matrix of the random forest binary classification model predicting presence (1) and absence (0).

Confusion matrix	Predicted: 0	Predicted: 1
Actual: 0	608	17
Actual: 1	4	663

### Presence/absence random forest model predictors

**Figure 3:**
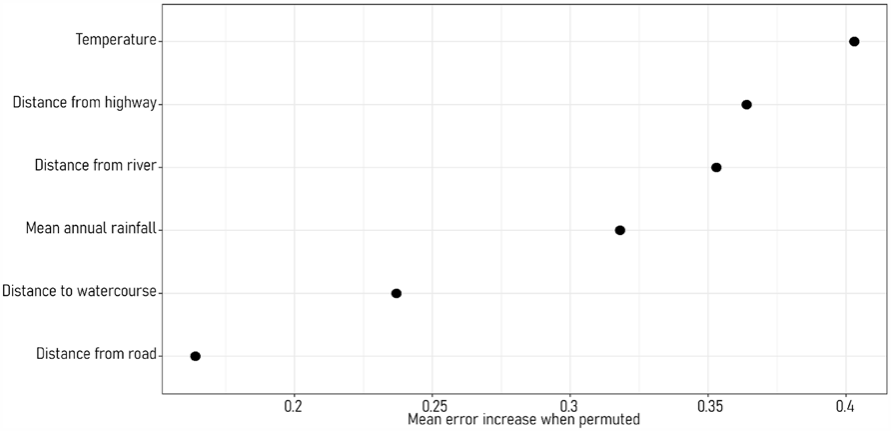
Variable importance plot showing the increase in mean error computed on the out-of-bag data across trees when predictors are permuted during the development of the random forest.

**Figure 4:**
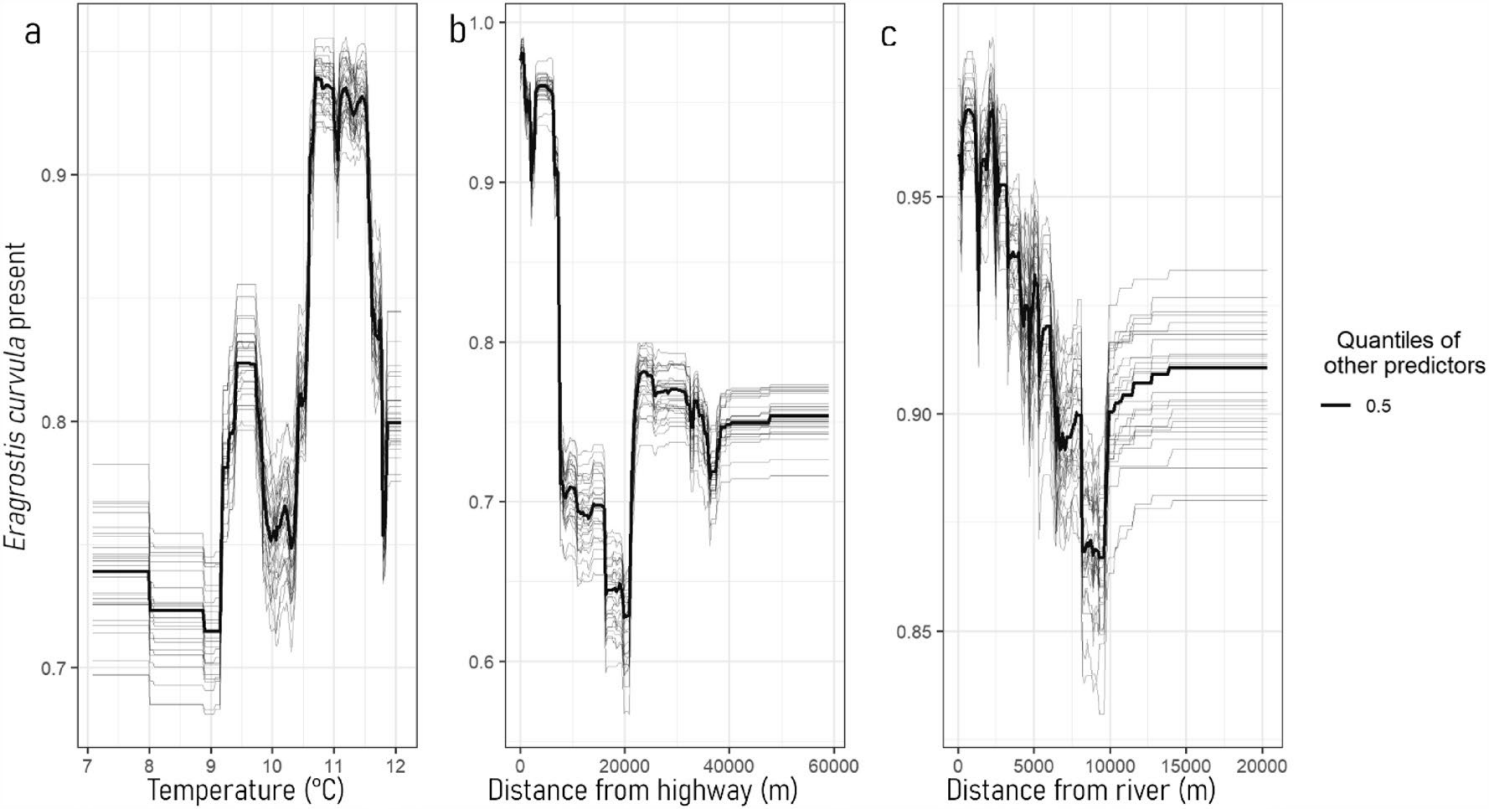
Response curves of the random forest model showing the top three important predictor variables derived from the model, (**a**) mean annual temperature (°C) 1976–2005, (**b**) distance from highway (m) and (**c**) distance from river (m). The bold black line indicates the response of *E. curvula* presence as a result of changes in the x-axis predictor variable, with all other predictor variables held at their 50% quantile. The thin grey lines show the variation of the response of *E. curvula* to the x-axis predictor variable after 30 repetitions.

All predictor variables had some influence on the RF P/A, with the removal of any one predictor increasing the mean error when permuted (Fig. 3). Temperature, distance from highway and distance from river were the most important predictor variables when minimising error in the RF P/A, respectively. The relationship between *E. curvula* and the three most important predictor variables does not appear linear when all other predictor variables are held constant, reflecting the complex interactions among predictor variables in predicting the distribution of *E. curvula* (Fig. 4). Variation in the response curve of the three most important predictor variables has minimal deviation from the general trend (Fig. 4).

## Discussion

Adopting a modelling approach to predicting the potential presence and absence of *E. curvula* has the potential to significantly benefit efforts to suppress its spread throughout the region by assisting targeted, proactive management strategies. Our model is empirically based on physical and ecological parameters offering further value in identifying potential mitigation strategies to reduce or contain its spread. The predictor variables used in the RF P/A were chosen to be a balance between easily accessible data available for public download and ecologically relevant variables that would likely impact the distribution of an invasive species^6,10^. As such, the observed influence of individual parameters (and their interactions) gives functional insight into potential mechanisms that may be used to constrain spread. Figure 4 highlights the complex non-linear relationship between temperature, distance from highway and distance from river and *E. curvula* presence. Roozbeh Valavi et al.^28^ conclude that flexible models, such as RF, can effectively predict a range of response variables in spatially distinct areas, such as those found in the SMRC. Roozbeh Valavi et al.^28^ define a flexible model as a modal that can adapt to simple and complex data, fitting closely to the data when needed but avoiding overfitting by not capturing random noise.

Tables 1 and 2 highlight high model performance, with a notable decrease in R squared (out of bag) compared to the other performance metrics. This notable decrease reflects the RF P/A limitations in modelling the presence or absence of *E. curvula* beyond the data it has been trained on. However, the RF P/A highlights the model’s explanatory power due to the high Kappa and F1 scores. Specifically, the RF P/A produces few false positives and fewer false negatives (Table 2). Few false negatives are preferred in this context, as under the precautionary principle, it would be considered better to assume the presence of an invasive species, check and be incorrect rather than believe that there are no invasive species in an area, risk being incorrect and the invasive species continuing to spread^51^. Using a similar workflow, De Simone et al.^52^ obtained an overall accuracy of 94.76% and a kappa of 88% when using RF for species distribution modelling of the invasive perennial grass *Brachypodium genuense* in an Italian grassland system*.* De Simone et al.^52^ find slope, elevation and solar radiation to be the most important predictor variable of B. genuense distribution, as opposed to temperature, distance from roads and distance from rivers in our research. Differentiating this research from that of De Simone et al.^52^ is that the selection the predictor variables used in this research were chosen based on their ecological relevance to *E. curvula’s* occurrence^6,10,53^. The predictor variables of De Simone et al.^52^ were chosen based on topographic variables available using the Sentinel-2 image database. Regardless, both De Simone et al.^52^ and this research highlight the utility of RF in developing accurate species distribution models for modelling invasive species within grassland ecosystems.

### Temperature as a predictor of *Eragrostis curvula*

The predictor variable of temperature was the most important variable in the RF P/A. Removing temperature as a variable from the model had the most detrimental effect on the RF P/A performance regarding error rate. Ngoy and Shebitz^20^ found a similar association between *E. curvula* and temperature, where the mean annual temperature was one of the most significant predictors of *E. curvula* across New Jersey in the United States of America. The ecological relevance of temperature to plant growth relates to the optimal conditions a plant requires for the most efficient metabolic processes^54,55^.

Temperature has been identified as an important predictor variable in the RF P/A, but a simple linear relationship cannot be assumed due to the nature of RFM. For example, the sample point located at longitude 148.57 and latitude −36.66 has an average annual temperature of 10.6 °C and has *E. curvula* present, while another sample point located at longitude 149.20 and latitude −36.29 also has an average annual temperature of 10.6 °C but does not have *E. curvula* present. Temperature in isolation is, therefore, not a reliable predictor in the RF P/A. However, when considered in the context of the other predictor variables, it becomes important in minimising error and maximising the accuracy of the RF P/A. Figure 4 highlights this complex relationship with no clear trend in the temperature response curve when all other predictor variables are held constant. A peak between 11 and 12 °C may indicate that *E. curvula* prefers this annual mean temperature within the study region. Roberts et al.^6,10^ found that temperatures between 17/7 °C for *E. curvula* seed collected from Maffra, Tenterfield, and Shepparton, Australia, germination time and survival rate were lower than at higher temperature ranges when investigating the germination biology for four different populations of *E. curvula*. Roberts et al.^6,10^ observed that temperature did not directly influence *E. curvula* seeds obtained from Midvale, Australia. The finding of Roberts et al.^6,10^ provides further evidence to support the phenotypic plasticity of *E. curvula* and its ability to adapt to a wide range of environmental conditions. Thus, making *E. curvula* a species well adapted to spatial dispersal across landscapes if given appropriate dispersal corridors such as roads and waterways.

### Roads as a predictor of *Eragrostis curvula*

The distance to highway was the next most important predictor variable in the RF P/A. Similar to temperature, due to the nature of RFM, the model does not make generalisations regarding the relationship between *E. curvula* and distance to highway. However, the ecological context suggests that as the distance from frequently used roads increases, the cover of invasive species such as *E. curvula* is likely to decrease, a trend supported by Fig. 4. Figure 4B shows a pattern of forecasting higher predictions of *E. curvula* present when the distance from highways is low, and all other predictor variables are constant. The distance to highway acts as a proxy to human dispersal and, by extension, a vector for *E. curvula* dispersal. Distance from human dispersal corridors, such as roads, consistently appears to influence invasive plant presence and density^16,28, 30,35,44, 56–58^. Gelbard and Belnap^59^ found that as road use increases, the cover and richness of invasive species also increase adjacent to these roads and decrease as distance increases from the road into the interior of the surrounding area. Hansen and Clevenger^60^ suggest that corridor edges, such as roads and railway lines, are more prone to invasion as they may act as microhabitats or microclimates suitable for invasion recruitment. These microclimates are suitable due to areas adjacent to roads often having barriers to invasion removed, such as competition for light and soil moisture^60^. The concept of ecosystem invasibility is relevant here as it pertains to roads acting as a vector of disturbance, thus removing competition and opening niche space for invasive species to exploit^61,62^. For this reason, areas adjacent to roads can be seen as more invasable, allowing invasive species to establish and spread into surrounding areas. Furthermore, roads can increase the propagule pressure of invasive plants where seeds are spread long distances by vehicle travel^63–65^.

### Rivers as a predictor of *Eragrostis curvula*

Rivers are a dynamic, inherently spatial system as water is transported through the landscape, with material actively or passively transported in conjunction^66^. The dynamic and spatial nature of riverine systems alleviates one of the major barriers to invasive species recruitment: the dispersal of propagules to sites outside of their native range^66,67^. Furthermore, riverine systems and the associated riparian systems undergo regular disturbance in the form of flooding and livestock watering^67^. Nakayama, Nishihiro, Kayaba, Muranaka, and Washitani^68^ investigated the potential for *E. curvula* to spread via riverine systems by comparing the fall velocity of *E. curvula* seeds with different sediment particle sizes. Nakayama et al.^68^ found that sediment particle size of < 0.25 mm, here considered fine sand, had a similar fall velocity to *E. curvula.* Furthermore*,* a positive correlation between fine sand and *E. curvula* seed occurred at the study site of the Kinu River, Japan. Nakayama et al.^68^ provide evidence for *E. curvula* to disperse via hydrochory and link to our findings that show a trend for lower *E. curvula* presence prediction as the distance from rivers increases.

### Limitations of the predicted presence and absence of *Eragrostis curvula*

Due to the large number of seeds *E. curvula* produces and its high germination rate, there will likely be other *E. curvula* plants within at least 1 hectare of any given plant^6,10^. Further, sites close to each other are likely to have more similar abiotic conditions than sites further away which is the basis of spatial interpolation^69^. Therefore, sites, where *E. curvula* was not observed when conducting the vegetation survey cannot be ruled out as incapable of supporting *E. curvula*, particularly if an *E. curvula* plant was observed within close proximity. Further, due to the relative course resolution of predictor variables of the climatic variable of temperature and rainfall, compared to the sampling spatial resolution, finer microclimatic variations are generalised over a larger area, potentially overlooking important fine scale influence on *E. curvula* distribution^70,71^. Despite several limitations to data collection imposed by COVID-19 travel restrictions and the Black Summer bushfires, our RF P/A showed value in outperforming the neutral random model and therefore has great potential to assist in targeted efforts for monitoring and managing *E. curuvla* presence. However, these limitations potentially influenced the availability and quality of data used to train the model due to the reliance on ancillary data, impacting the reliability of predictions.

Further, due to the intentional decision to use open-source, free-available data as predictor variables, there are inconsistencies in the spatial resolution of the data. Mishra et al.^72^ show that increasing spatial resolution can result in higher classification rates of land use and land cover classes. The inconsistencies in the spatial resolution of the predictor variables could overinflate the importance of certain predictor variables. However, there is limited research on this as a factor influencing bias.

## Conclusion

The predictor variables of annual mean temperature, distance from highway and distance from river were identified as the most important variables predicting the presence of *E. curvula* in the study area. These predictor variables align with previously identified predictor variables of *E. curvula* and other invasive plants relating to hydrochory and zoochory^6,10^. The RF P/A indicates conditions in which the presence of *E. curvula* is supported based on a range of open-access landscape predictor variables. However, the model’s performance metrics based on extrapolating outside of the data it has been trained on should be interpreted cautiously. As such, the outcomes of our findings should be used as a tool as part of a larger toolbox rather than a rule used to manage *E. curvula* as part of integrated management strategies. However, the model has inherent limitations in high-resolution accuracy due to the decision to use easily accessible predictor variables with differing spatial resolutions. As more high resolution becomes freely available, further predictive models will utilise this and likely develop more precise and accurate models.

Furthermore, the RF P/A should be tested on data it has not been trained on and compare ground-truth observational data with predictions generated by the RF P/A. Regardless of how accurate predictive models become, community communication is necessary so properties without *E. curvula* neighbouring properties with *E. curvula* can act accordingly to put management strategies in place to minimise the risk of further spread, and the outcome from the RF P/A can be utilised to inform those at most risk. The RF P/A produced may aid community members in understanding the possible extent of *E. curvula* based on their location in the landscape and highlight the areas predicted to be the most susceptible to *E. curvula* presence. This research highlights the necessity for more detailed mapping of the current distribution and density of *E. curvula* across the Snowy Monaro to prevent the spreading from high-density *E. curvula* areas to low-density *E. curvula* areas. If such a map existed in conjunction with the provided output from the RF P/A, then concentrated survey and management efforts could be focused on the most at-risk areas.

### Supplementary Information

Supplementary Information.

## Data Availability

The datasets generated during and/or analysed during the current study are available from the corresponding author on reasonable request.
